# Burden of Pediatric SARS-CoV-2 Hospitalizations during the Omicron Wave in Germany

**DOI:** 10.3390/v14102102

**Published:** 2022-09-22

**Authors:** Maren Doenhardt, Christin Gano, Anna-Lisa Sorg, Natalie Diffloth, Tobias Tenenbaum, Rüdiger von Kries, Reinhard Berner, Jakob P. Armann

**Affiliations:** 1 Department of Pediatrics, University Hospital and Medical Faculty Carl Gustav Carus, Technische Universität Dresden, 01307 Dresden, Germany; 2 Division of Pediatric Epidemiology, Institute of Social Pediatrics and Adolescent Medicine, Ludwig-Maximilians-University Munich, 80336 München, Germany; 3 Clinic for Child and Adolescent Medicine, Sana Klinikum Lichtenberg, Academic Teaching Hospital, Charité-Universitätsmedizin Berlin, 10365 Berlin, Germany

**Keywords:** SARS-CoV-2, COVID-19, Omicron, children, hospitalization

## Abstract

(1) Background: When the Omicron variant of SARS-CoV-2 first emerged in Germany in January 2022, data on related disease severity among children and adolescents were not yet available. Given Omicron’s high transmissibility, the ability to assess its impact on admission and hospitalization rates in children’s hospitals is critical for the purpose of understanding the scope of its burden on the German healthcare system. (2) Methods: From 24 January 2022 to 31 July 2022, SARS-CoV-2 cases admitted to German pediatric hospitals were monitored via a national, clinician-led reporting system (CLRS) established by the German Society for Pediatric Infectious Diseases (DGPI). Cases treated on general wards and intensive care units, as well as patient age and the need for respiratory support, were recorded. (3) Results: From January to July 2022, a median of 1.7 cases (range 0.4–3) per reporting pediatric hospital per day was hospitalized in general wards, whereas a median of 0.1 cases (range 0–0.4 cases) was admitted to intensive care units. Of all hospitalized patients, 4.2% received respiratory support. (4) Conclusions: Despite the high incidence rates documented in connection with the Omicron variant in early 2022, the number of pediatric hospital admissions, and especially the number of cases with the need for intensive care treatment and respiratory support due to symptomatic SARS-CoV-2 infection, remained relatively low. Higher Omicron incidence rates had only a modest impact on SARS-CoV-2-related admissions and hospitalization in German children’s hospitals.

## 1. Introduction

With the emergence of the Omicron variant of SARS-CoV-2 in January 2022, disease severity was widely reported to be lower than with previous variants [1,2]. Although severe cases of acute COVID-19 in children and adolescents generally appear to be rare [3], to date, little data regarding disease severity, specifically due to the Omicron variant, have been available. Given Omicron’s high transmissibility and relatedly high case numbers, even a low-risk variant has the possibility of leading to increased hospitalizations, thereby additionally burdening the healthcare system. 

Employing a clinician-led reporting system (CLRS) established by the German Society for Pediatric Infectious Diseases (DGPI), one initially designed to capture hospital admissions due to Respiratory Syncytial Virus (RSV) infections in the fall/winter season of 2021/2022 [4], we analyzed data on hospital admissions and severe disease courses in children and adolescents with confirmed SARS-CoV-2 infections in German pediatric hospitals during the Omicron wave [5].

## 2. Materials and Methods

The CLRS we used is a publicly accessible resource using the REDCap (Research Electronic Data Capture) platform hosted at the Technische Universität Dresden, Germany. All data on pediatric SARS-CoV-2 infections, related respiratory support, and hospitalization rates captured through this vehicle also were published and publicly accessible via the DGPI website [5]. The CLRS was promoted via the websites of the DGPI and the German Society of Pediatrics (DGKJ) and, additionally, was announced in a newsletter sent to all German pediatric hospitals. From 24 January 2022 to 31 July 2022, participating hospitals submitted data on children and adolescents with confirmed SARS-CoV-2 infections who had been admitted to these hospitals. SARS-CoV-2-positive admissions and cumulative cases in general pediatric and intensive care wards were documented daily. Cases reported were used to calculate average case numbers per reporting hospital per day. Patient age and the type of respiratory support received (invasive vs. non-invasive), as well as whether the patients had received any SARS-CoV-2-specific medical treatments during their hospital stays, were recorded. Incidence rates were extracted from the weekly report by the Robert Koch Institute, Berlin, Germany [6].

Statistical analysis: Linear regression models were used to analyze the association between incidence rates and outcomes of interest. These included the average number of admissions per reporting hospital per day, average daily hospitalizations per reporting hospital, the average number of inpatients in intensive care units (ICU) per reporting hospital per day, and the average number of patients on mechanical ventilation per reporting hospital per day. Statistics were calculated using SAS, version 9.4 (SAS Institute Inc., Cary, NC, USA).

## 3. Results

**Figure 1:**
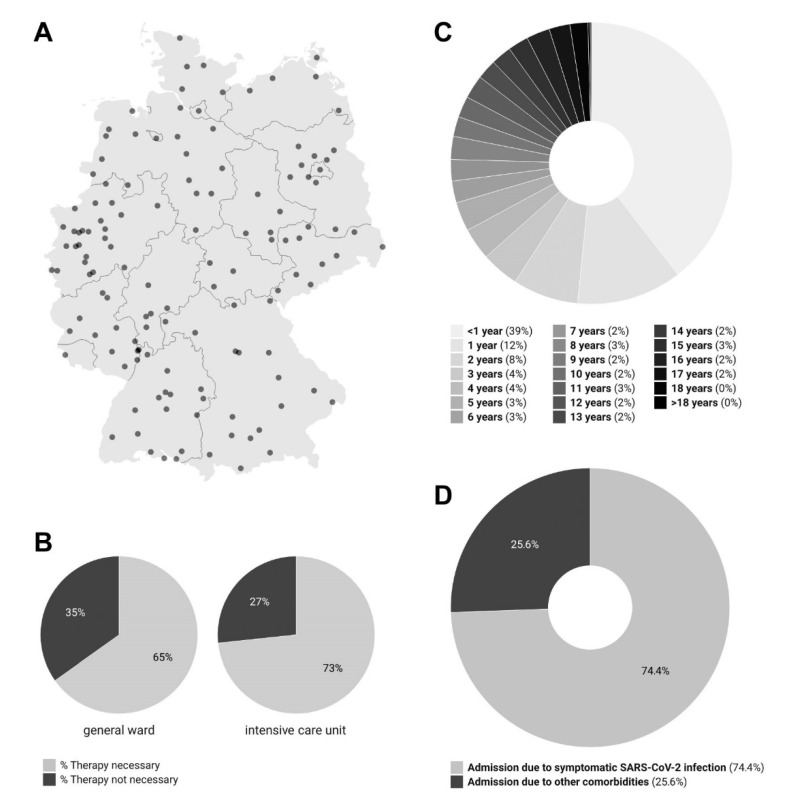
(**A**) German hospitals participating in the SARS-CoV-2 CLRS: Participation rate was independent of hospital size. (**B**) Need for SARS-CoV-2-specific therapies on general wards vs. intensive care units. (**C**) Age distribution of hospitalized SARS-CoV-2 cases. (**D**) Hospital admission due to symptomatic SARS-CoV-2 infections. All graphics refer to hospitalized children and adolescents with SARS-CoV-2 in Germany, as reported to the DGPI’s ad hoc SARS-CoV-2 register from 24 January 2022, to 31 July 2022.

With representation coming from all German states, municipal and university hospitals from 135 cities participated in the CLRS. Both small and large hospitals were included in the register (Figure 1A). At their peak, 89 out of 334 German pediatric hospitals (26.7%; 28 January 2022) participated in the CLRS; the minimum number was 7 (2.1%; 31 July 2022) [median of reporting hospitals: 35 (10.5%)]. 

An average of 177 hospitalized, pediatric, SARS-CoV-2 cases per day were reported in January, with a peak of 239 absolute cases per day on 8 February 2022. In May 2022, the average case number per day decreased to 30, the low point, and slightly increased to an average of 46 per day in July (Table S1). Given the varying hospital participation rates over time, average cases per reporting hospital per day were calculated in order to improve comparability. 

**Figure 2:**
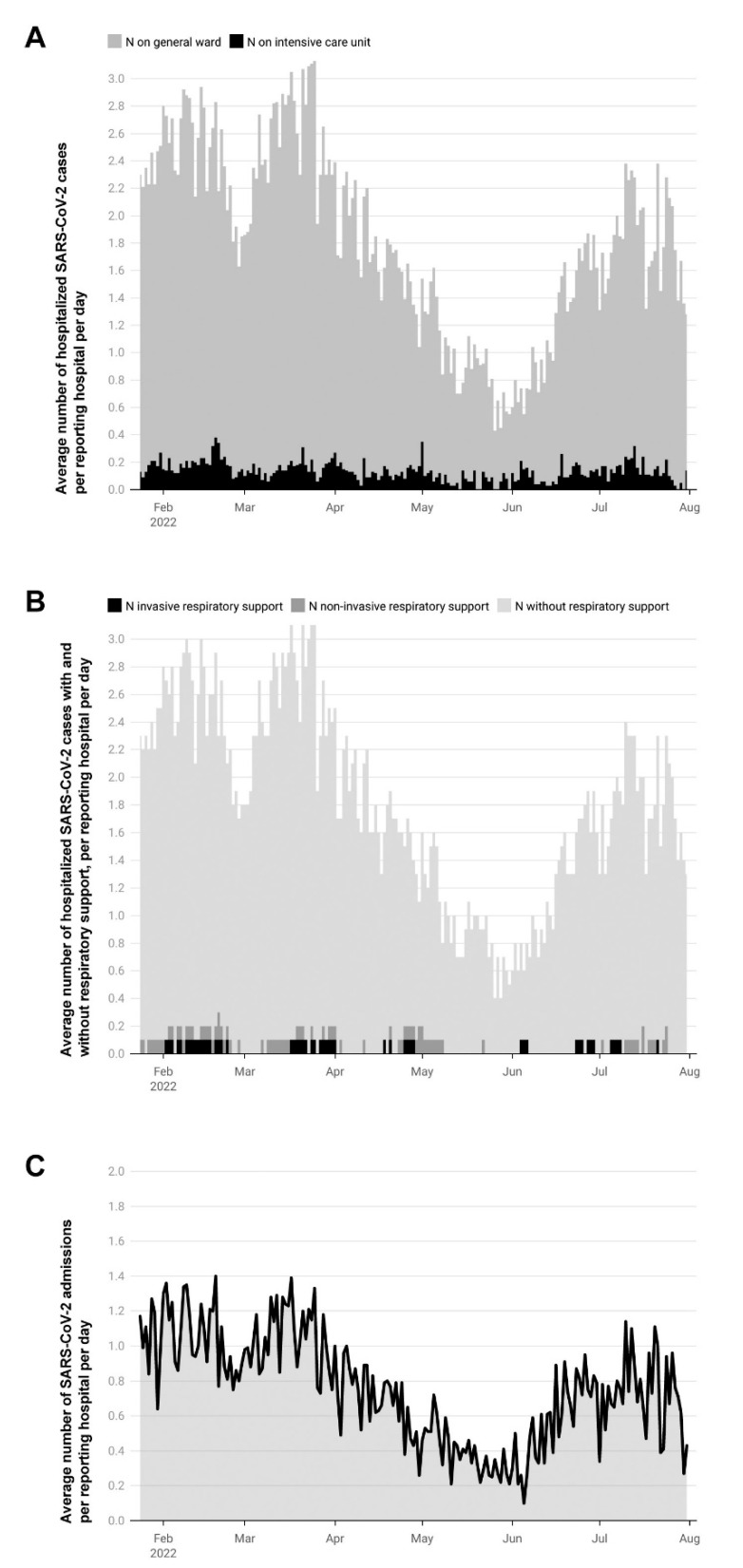
(**A**) Average number of hospitalized SARS-CoV-2 cases per reporting hospital per day. General wards and intensive care units are displayed separately. (**B**) Average number of hospitalized SARS-CoV-2 cases with and without respiratory support, per reporting hospital per day. (**C**) Average number of SARS-CoV-2 admissions per reporting hospital per day. All charts refer to hospitalized children and adolescents with SARS-CoV-2 in Germany, as reported to the DGPI’s ad hoc SARS-CoV-2 register from 24 January 2022, to 31 July 2022.

During the monitoring period, a median of 1.7 cases (range 0.4–3 cases) per reporting hospital per day was recorded on general wards, while a median of 0.1 cases (range 0–0.4 cases) per reporting hospital per day was reported on intensive care units (Figure 2A). The vast majority of patients did not require respiratory support (N = 15,049; 95.8%). Of those receiving respiratory support (N = 661; 4.2%), 2.4% (N = 386) were provided non-invasive support, such as continuous positive airway pressure (CPAP), non-invasive ventilation (NIV), or high-flow therapy; only 1.8% (N = 275) required invasive ventilation (Figure 2B). 

A preponderance of newly admitted patients was under two years old (N = 3435; 51%), with 39% (N = 2629) being <1 year old (Figure 1C). In 26% (N = 1591) of newly admitted cases, hospital admission was not directly related to the SARS-CoV-2 infection (Figure 1D), but rather due to other essential comorbidities. In general wards, 35% (N = 4213) of patients required no treatment for COVID-19; in the ICU, this number was 27% (N = 267) (Figure 1B). To date, the highest SARS-CoV-2 incidence rates in Germany have coincided with the Omicron wave, with cases peaking at 1758 per 100,000 on 26 March 2022. The incidence rate then decreased to its low point of 189 at the end of May. After this, the rate increased again to over 700 per 100,000 during the BA.4/BA.5 Omicron wave at the end of June [6]. Using linear regression analyses, daily average hospitalization, admissions, inpatient intensive care treatment, and invasive ventilation were found to be significantly associated with the SARS-CoV-2 incidence rate (Table 1 and Supplemental Figure S1). An increase in the incidence rate of 100 per 100,000 SARS-CoV-2 infections was significantly correlated with an increase in the expected daily average number of hospitalizations (+0.094), admissions (+0.025), ICU treatments (+0.0073), and patients requiring invasive ventilation (+0.0065).

## 4. Discussion

During the Omicron wave, approximately 10% of German pediatric hospitals contributed data to the CLRS—a participation level allowing us to report with confidence regarding the SARS-CoV-2-related burden on the German pediatric healthcare system. Despite the highest incidence of SARS-CoV-2 in Germany having been recorded during our study period, pediatric admissions and hospitalization nevertheless remained consistently low, with the maximum number of new admissions per reporting pediatric hospital per day being 1.4 on March 17, 2022, and the maximum number of SARS-CoV-2 positive hospitalizations being 3.13 per reporting pediatric hospital per day on March 25, 2022 (Figure 2). The median of hospitalized SARS-CoV-2 cases per reporting hospital per day (median 1.8, range 0.4–3.1) was only slightly higher than the median of newly admitted cases per reporting hospital per day (median 1.0, range 0.5–1.4). This indicates that the average length of stay did not exceed 48 h and further suggests individual disease severity generally has been mild.

During the study period overall, only a small number of patients required either ICU treatment (N = 1129; 7.2%) or invasive ventilation (N = 275; 1.8%). Given the overall lower disease severity in connection with the Omicron wave, pediatric hospitals in Germany were not significantly additionally burdened.

In addition, 26% (N = 1591) of children and adolescents with confirmed SARS-CoV-2 infections were admitted for reasons unrelated to this infection (Figure 1D). Other studies have had similar findings regarding incidentally positively tested patients [7]. For this reason, cases falling into this category should be excluded from analysis when evaluating the SARS-CoV-2-related burden on hospital systems.

By contrast, data collected from the same CLRS during fall/winter 2021–2022—at that point, in order to capture RSV-associated hospitalizations and disease severity in pediatric hospitals in Germany—showed two to three times higher rates of hospitalization per pediatric hospital per day, as well as a significantly higher need for respiratory support (RSV 2794/24,331; 11.5% vs. SARS-CoV-2 661/15,710; 4.2%). The ICU admission rates during the RSV wave in November-December 2021 (2105/24,331; 8.7%) were similar to those during the SARS-CoV-2 Omicron wave (1129/15,710; 7.2%) [4].

This difference in disease burden between SARS-CoV-2 and RSV is even more pronounced when considering that RSV only affects a very limited age group and that there is no universal screening for RSV, a leading cause of underestimating the true disease burden. 

While some data have shown Omicron to lead to higher morbidity among unvaccinated and/or previously uninfected children as compared to influenza/parainfluenza infections [8], our register did not include data regarding vaccination status, prior COVID-19 disease, and/or comorbidities. According to Edwards et al., Omicron is independently associated with ICU admission and severe disease, as defined by the WHO Clinical Progression Scale in children without SARS-CoV-2 immunization or prior COVID-19 infection [9]. Vaccination provides moderate protection against symptomatic and severe Omicron infection in children [10,11,12]. For this reason, it has a positive impact on hospitalization rates in previously uninfected children [13]. However, in the aftermath of the Omicron wave, along with the launch of pediatric COVID-19 vaccination campaigns in Germany, the remaining number of immune-naïve children has become minimal [14]. On this basis, we anticipate that an Omicron wave in fall 2022 is even less likely to pose a significant burden on the hospital system than did the previous wave. 

SARS-CoV-2-related admissions and hospitalization have been shown to increase along with a rise in incidence rates in the general population. However, this effect is quite modest, with only +0.025 admissions, +0.094 hospitalizations, and +0.0073 treatment in ICU and +0.0065 patients requiring invasive ventilation per hospital per day for each increase in the incidence rate by 100 per 100,000. Based on currently available data, we anticipate that this effect will not pose significant challenges to children’s hospitals during future SARS-CoV-2 waves, regardless of actual case numbers.

Our study is limited by the register’s design. Given its focus on the burden on pediatric hospitals via the measurement of SARS-CoV-2 hospital admissions and hospitalization rates over time, individual parameters such as clinical appearance, risk factors, or vaccination status cannot be evaluated. Furthermore, given the voluntary nature of the CLRS, we are unable to provide information on hospitals that chose not to participate vs. those that did. This also might lead to a degree of reporting and selection bias. Nevertheless, with up to one-quarter of all pediatric hospitals reporting to the CLRS, we are confident that the general trends observed can be considered reliable. 

## 5. Conclusions

Our CLRS provides a vital tool for healthcare professionals and national policymakers when assessing, evaluating, and allocating pediatric hospital care resources. High participation rates among hospitals, as well as the quality of data submitted, are essential to the value the CLRS is able to deliver. With an eye toward the upcoming fall/winter season, the CLRS was updated and amended in August 2022. Several airway pathogens, (e.g., SARS-CoV-2, RSV, and Influenza), will now be included as part of the same data query.

## Figures and Tables

**Table 1:** Regression models for different outcome parameters: Relationship between the daily average numbers of hospitalizations, admissions, inpatient treatment in ICU, and patients requiring invasive ventilation and the SARS-CoV-2 incidence rates among children and adolescents in Germany.

Outcomes of Interest: Average per Hospital per Day	Linear Regression Coefficient β (95% CI)	Standard Error (SE)	Effect of an Increase in the SARS-CoV-2 Incidence Rate by 100 per 100,000	*p*-Value
Hospitalization	0.00094 (0.00061, 0.00127)	0.00017	+0.094	<0.0001
Admissions	0.00025 (0.000053, 0.00045)	0.000099	+0.025	0.0136
Inpatient intensive care treatment	0.000073 (0.000015, 0.00013)	0.000029	+0.0073	0.0148
Invasive ventilation	0.000065 (0.000043, 0.000087)	0.000011	+0.0065	<0.0001

## Data Availability

Not applicable.

## Supplementary Materials

The following supporting information may be downloaded at: https://www.mdpi.com/article/10.3390/v14102102/s1, Table S1: Average number of reporting hospitals and SARS-CoV-2 cases from 24 January 2022, to 31 July 2022, Figure S1. Association of SARS-CoV-2 incidence rates and (A) average hospitalizations per reporting hospital per day, (B) average admissions per reporting hospital per day, (C) average number of inpatient intensive care treatments per reporting hospital per day, and (D) average number of ventilated patients per reporting hospital per day.
