# Supplementary material for: Burden of Pediatric SARS-CoV-2 Hospitalizations during the Omicron Wave in Germany
Source: Viruses. 2022 Sep 22;14(10):2102. doi: 10.3390/v14102102 (PMC9607436; doi:10.3390/v14102102)
Supplement: Supplementary file 1 [file viruses-14-02102-s001.zip › viruses-1914404-supplementary.pdf]

Supplemental Table S1. Average number of reporting hospitals and SARS-CoV-2 cases from January 24, 2022 - July 31, 2022.

	January	February	March	April	May	June	July
Number of reporting pediatric hospitals	76	66	53	38	31	28	25
% of reporting pediatric hospitals in Germany*	22.8	19.8	15.9	11.4	9.3	8.4	7.5
Number of patients on general wards	164	153	127	64	28	31	43
Number of patients on intensive care units	13	13	8	5	2	3	3
Total number of patients hospitalized	177	166	135	69	30	34	46

*The total number of German pediatric hospitals used as a basis for calculating percentages was 334.

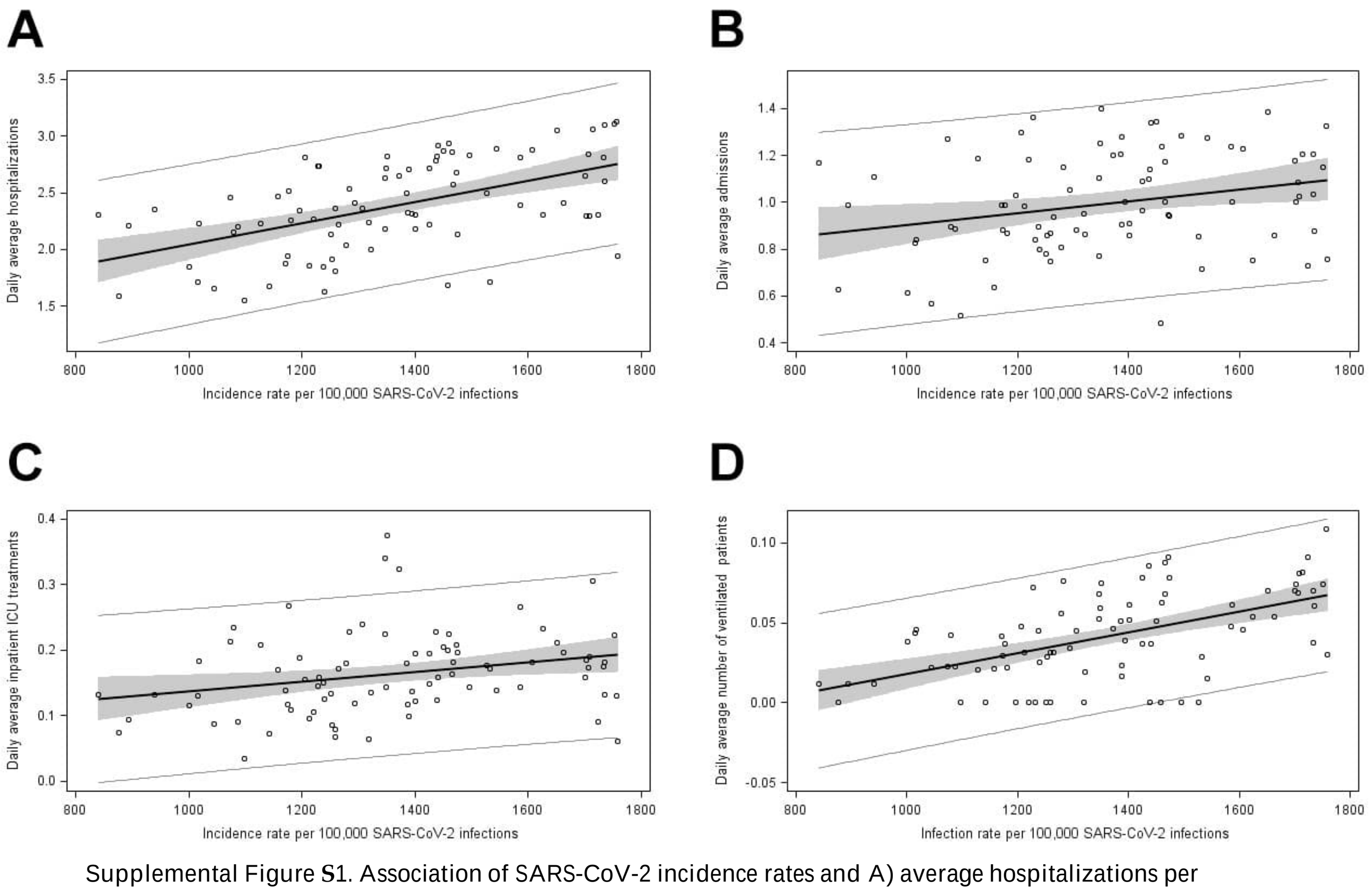

Supplemental Figure S1. Association of SARS-CoV-2 incidence rates and A) average hospitalizations per reporting hospital per day, B) average admissions per reporting hospital per day, C) average number of inpatient intensive care treatments per reporting hospital per day and D) average number of ventilated patients per reporting hospital per day.
